# Molecular detection of *Trypanosoma* spp. and *Hepatocystis* parasite infections of bats in Northern Nigeria

**DOI:** 10.1017/S0031182022000890

**Published:** 2022-09

**Authors:** J. Kamani, Y. J. Atuman, D. A. Oche, A. Shekaro, O. Werb, I. Ejotre, J. Schaer

**Affiliations:** 1 Parasitology Division, National Veterinary Research Institute (NVRI), PMB 01, Vom, Plateau State, Nigeria; 2 Animal Health & Production, Akperan Orshi Polytechnic Yandev, Gboko, Benue State, Nigeria; 3 Department of Molecular Parasitology, Institute of Biology, Humboldt University, Berlin, Germany; 4 Department of Biology, Muni University, Arua, Uganda

**Keywords:** Bats, Chiroptera, Haemosporida, *Hepatocystis*, *Trypanosoma*

## Abstract

Bats are mammalian hosts to a large diversity of eukaryotic protozoan blood parasites, including different genera of haemosporidians and diverse species of trypanosomes. Phylogenetic studies suggest that bats, particularly in Africa, have played an important role in the evolutionary histories of these parasite groups. However, our understanding of the diversity and distribution of chiropteran haemosporidians and trypanosomes in Africa remains tenuous. We investigated the prevalence and phylogenetic relationships of the blood parasites in different bat species in Northern Nigeria using molecular methods. A low prevalence of *Hepatocystis* parasites was detected in a potentially rare host species, the African straw-coloured fruit bat (*Eidolon helvum*) confirming yet another fruit bat species in the diverse range of African bat hosts. Trypanosome infections were identified in 3 different bat species. The trypanosomes of *Mops* cf. *pumilus* were recovered as a distinct lineage that is related to *Trypanosoma erneyi*, a species which is closely related to *Trypanosoma dionisii* and *Trypanosoma cruzi. Nycteris* cf. *macrotis* bats were infected with trypanosomes that are related to the distinct lineage of *Trypanosoma* cf. *livingstonei* parasites. Further, 2 different lineages of trypanosomes in *E. helvum* bats share highest nucleotide identities with *Trypanosoma livingstonei* and a group of *Trypanosoma* sp. parasites that are closely related to *T.* cf. *livingstonei* and *T. livingstonei*, respectively. The findings of this study confirm the notion that trypanosomes of African bats are phylogenetically diverse and that African bats might harbour a variety of yet undescribed trypanosome species.

## Graphical abstract

**Figure:**
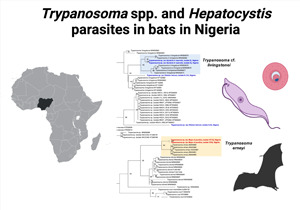

## Introduction

Bats, a species-rich group with over 1454 species (Simmons, 2005; Miller-Butterworth *et al*., 2007; Lack *et al*., 2010) are mammalian hosts to a large diversity of eukaryotic protozoan blood parasites. These comprise different genera of haemosporidian parasites and diverse species of trypanosomes (e.g. Lima *et al*., 2013; Schaer *et al*., 2013; Clement *et al*., 2020).

The apicomplexan parasites of the order Haemosporida infect diverse vertebrate hosts, comprising mammals, squamates and birds and use different dipteran hosts as vectors (Garnham, 1966; Levine, 1988). The over 500 described haemosporidian parasites are classified in the genus *Plasmodium* that includes the human-infecting species that cause the malaria disease, plus 9 additional genera (Martinsen and Perkins, 2013). Among mammals, bats harbour the most diverse set of haemosporidian parasites and results of previous phylogenetic studies suggest that bats have played an important role in the evolutionary history of haemosporidian parasites (Duval *et al*., 2007; Schaer *et al*., 2013; Perkins and Schaer, 2016; Galen *et al*., 2018).

The kinetoplastid flagellated *Trypanosoma* parasites feature complex lifecycles and are mainly transmitted to the vertebrate host by haematophagous arthropods or leeches (Simpson *et al*., 2006). In Africa, the *Trypanosoma* species *Trypanosoma brucei* sensu lato, *Trypanosoma vivax* and *Trypanosoma congolense* present a threat to both humans and livestock (Morrison *et al*., 2016; Büscher *et al*., 2017). Previous studies have identified a high diversity of *Trypanosoma* species in bats and revealed that bats also played an important role in the evolutionary history of these parasites (e.g. Hamilton *et al*., 2012; Lima *et al*., 2012, 2013; Clement *et al*., 2020; Austen and Barbosa, 2021). The investigation of the diversity, distribution and phylogenetic relationships of trypanosome species of wildlife, including bats, is essential for a better understanding of the evolution of the whole parasite group.

With about 85 species of bats, Nigeria represents a diversity hotspot in Africa (Simmons and Cirranello, 2022). However, the knowledge about haemosporidian parasites and trypanosomes in Nigerian bats is scarce (Atama *et al*., 2019). Here, we investigated the prevalence and phylogenetic relationships of haemosporidian and trypanosome parasites in different bat species in Northern Nigeria using molecular methods.

## Materials and methods

**Fig. 1.**
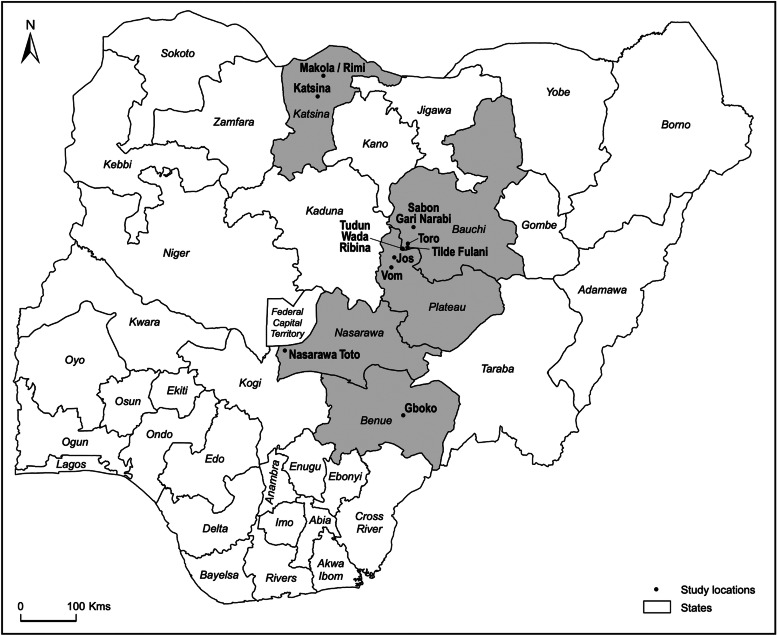
Map of Nigeria. Study areas are highlighted (in grey) and sampling sites are depicted.

Bats were sampled in Northern Nigeria between December 2018 and March 2019 in the states of Bauchi, Benue, Katsina, Nasarawa and Plateau. The sampling sites comprised 10 peri-domestic roosting sites (trees, caves, ceiling/eaves of residential buildings) (Fig. 1).

### Field sampling and microscopy

Bats were captured using improvised traps produced from fishing nets and wooden poles. The nets were set every capture night in some distance to their roosting sites. The bats were gently removed from the nets and transferred into individual bags and genus and/or species were identified following the keys available in Patterson and Webala (Patterson and Webala, 2012). Bats were captured and sacrificed within the scope of a larger project focusing on viral and bacterial infections in bats (e.g. Kamani *et al*., 2021). The bats were anaesthetized using a combination of 5 mg kg^−1^ ketamine plus 2 mg kg^−1^ of xylazine injected intramuscularly. The voucher specimens were preserved in 70% (vol vol^−1^) ethanol and stored in the Parasitology Division NVRI Vom, in Nigeria. Blood samples were collected by venous puncture of the brachial vein and collected in capillary tubes. For the investigation of blood parasites, blood samples were used to spot blood dots onto DNA FTA cards (Whatman) and to prepare thin blood smears on slides. The blood smears were dried and fixed in 99–100% (vol vol^−1^) methanol solution for 3 s and later stained with 10% Giemsa solution for 50 min. Giemsa-stained blood smears were examined for the presence of haemosporidian and trypanosome parasites using light microscopy at a magnification of ×1000 with immersion oil.

### Molecular methods

Whole genomic DNA was extracted from the dried blood dots on DNA FTA cards using the DNeasy extraction kit (Qiagen, Hilden, Germany) (e.g. Schaer *et al*., 2017). The protocol for animal tissues was performed and samples were eluted in 60 *μ*L AE buffer. Polymerase chain reactions (PCRs) were performed using the AllTaq Master Mix Kit (QIAGEN) with 4–5 *μ*L of genomic DNA as the template, and 1 *μ*L of each primer (10 mm).

To confirm morphological bat identifications, most of the samples were genotyped using 1 or more of the following genetic markers for the bat hosts: the mitochondrial cytochrome b (*cytb*) and the nuclear introns Acyl-CoA oxidase 2, intron 3 (*acox2*), and Beta-fibrinogen gene, intron 7 (*fgb*) (Table S1). A part of the *cytb* gene was sequenced for 47 of the 60 *E. helvum* individuals, which featured 100% nucleotide identity with *E. helvum* reference sequences in NCBI GenBank. For 11 *E. helvum* individuals, 650 bp of the *fgb* were additionally or instead sequenced, which again resulted in a nucleotide identity of 100% with published *E. helvum* reference sequences. To identify 3 individuals that were morphologically identified as *Tadarida* sp. to species level, *cytb* sequences were generated which resulted in highest nucleotide identities of 99.2% with reference sequences of *Mops condylurus* (e.g. GenBank accession number EF474030) and thus might represent *M. condylurus* or a close related species and are therefore termed *M*. cf. *condylurus*. Five out of 8 individuals that were morphologically identified as *Chaerephon* sp. were genotyped with *cytb* (featuring identical *cytb* sequences) and highest nucleotide identities of 98.4% were recovered with reference sequences for *Mops pumilus* (e.g. GQ489157). Again, these bats might represent the species *M. pumilus* or a close related species and are termed *M*. cf. *pumilus*. Twelve out of 16 *Nycteris* sp. individuals were genotyped with *cytb* and highest nucleotide identities of 94.7% (sample number KJ-M), 96.98% (sample numbers KJ-B, KJ-C, KJ-D, KJ-J) and 97.3% (remaining samples) were recovered with the reference sequence for *Nycteris macrotis* (accession number MT586790). The sequences of the nuclear *acox2* for 2 of the *Nycteris* sp. samples (samples numbers KJ-E, KJ-F) featured a 98.7% nucleotide identity with the reference sequence for *Nycteris macrotis* complex sp.1 (MK837356). We, therefore, tentatively refer to the individuals of the study as *Nycteris* cf. *macrotis.*

The mitochondrial cytochrome b (*cytb*) gene was targeted for detection and subsequent phylogenetic analysis of haemosporidian parasites (e.g. Schaer *et al*., 2013). Four genes were used for molecular detection of haemosporidian parasites: the mitochondrial genes cytochrome b (*cytb*) and cytochrome oxidase 1 (*cox1*); the apicoplast caseinolytic protease gene (*clpC*); and the nuclear gene elongation factor 2 (*ef2*) (Table S1) (Schaer *et al*., 2013).

For the detection of trypanosomes, a nested-PCR approach was carried out for the amplification of the partial sequence of about 640 bp of the small subunit 18S ribosomal RNA gene (18S rRNA) following Noyes *et al*. (1999). A second gene, the nuclear glycosomal glyceraldehyde phosphate dehydrogenase (*gGAPDH*), was subsequently targeted for all positive samples in a nested-PCR approach following Clement *et al*. (2020). All primers are listed in Table S1. All positive PCR products were sequenced with the amplification primers and run on an ABI-373 sequencer. The software Geneious Prime 2022.1.1 (https://www.geneious.com) was used to quality-check and manually edit all nucleotide sequences. Ambiguous base calls or missing data were coded with N's or the corresponding ambiguity code. Sequences were aligned using the MAFFT algorithm (Katoh *et al*., 2002; Katoh and Standley, 2013). Sequences for the analysis of the phylogenetic relationships of *Hepatocystis* comprised 531 nucleotides (nt) of the partial *cytb* gene. Sequence alignments for the analysis of *Trypanosoma* spp. included 1022 nt for 18S rRNA and 894 nt for *gGAPDH*, respectively. Reference sequences were retrieved from GenBank and added to the alignments (all accession numbers for analysis of *Hepatocystis* parasites are listed in Table S2, all accession numbers for 18S rRNA and *gGAPDH* analyses are listed in the corresponding phylogenetic tree figures and Table S3). The software ModelTest-NG was used to test different DNA substitution models (Darriba *et al*., 2020). Phylogenetic relationships were evaluated with maximum likelihood (ML) analysis and carried out in the software raxmlGUI version 2.0.6 (Edler *et al*., 2021). The *Hepatocystis* analysis of the partial *cytb* was carried out using the substitution model GTR + I (proportion of invariant sites) + Gamma (rate heterogeneity) and the taxon *Leucocytozoon* as outgroup. Nodal support was evaluated using 1000 replicates (thorough bootstrap). The trypanosome ML analysis of the 18S rRNA and *gGAPDH* gene were carried out using the models TIM3 + I + G and GTR + I + G, respectively, and 10 000 replicates and *Trypanosoma lewisi* as outgroup taxon. Phylogenetic trees were displayed in FigTree (http://tree.bio.ed.ac.uk/software/figtree/).

## Results

A total of 95 bats belonging to 3 bat families were investigated. The greater part of the captured bats (*n* = 68) belonged to the African fruit bat species *Eidolon helvum*. Further, 16 *Nycteris* cf. *macrotis* of the family Nycteridae, 8 *Mops* cf. *pumilus* and 3 *Mops* cf. *condylurus* (Molossidae) were screened for haemosporidian parasite and trypanosome parasite infections (Table 1).

**Table 1.** Investigated bat species and prevalences of haemosporidian and trypanosome parasite infections

Bat species (Bat family)	Total number of individuals	Haemosporidan parasite prevalence (%)	*Trypanosoma* sp. prevalence (%)
*Mops* cf. *condylurus* (Molossidae)	3	0/3 (0)	0/3 (0)
*Mops* cf. *pumilus* (Molossidae)	8	0/8 (0)	3/8 (37.5)
*Nycteris* cf. *macrotis* (Nycteridae)	16	0/16 (0)	11/16 (68.8)
*Eidolon helvum* (Pteropodidae)	68	4/68 (5.9)	2/68 (2.9)
Total	95	4/95 (4.2)	16/95 (16.8)

### Prevalence and phylogenetic characterization of haemosporidian parasites

**Fig. 2.**
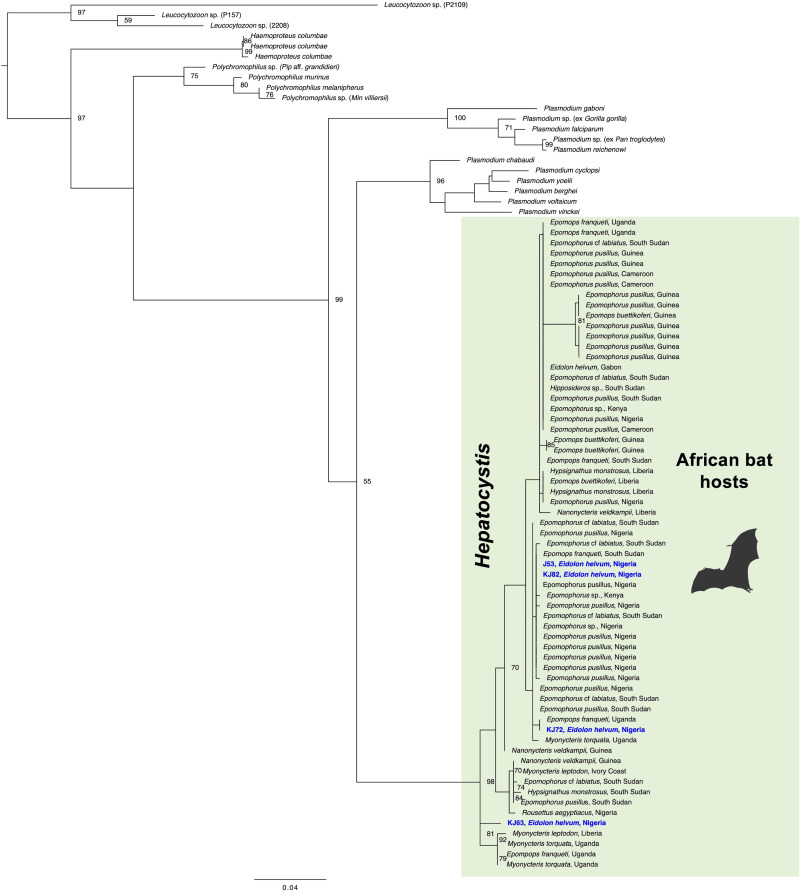
Maximum likelihood analysis of *Hepatocystis* parasites in African bats. The analysis is based on the mitochondrial gene *cytb* (1119 bp) and was run in the context of the major haemosporidian parasite clades comprising *Leucocytozoon* (used as outgroup taxon), *Haemoproteus, Polychromophilus* and the mammalian-infecting *Plasmodium* clades *Plasmodium* (*Laverania*) and *Plasmodium* (*Vinckeia*), with the latter representing the closest relative to *Hepatocystis* parasites. The phylogenetic analysis recovered the *Hepatocystis* sequences from *E. helvum* within the African bat *Hepatocystis* clade without any apparent geographic or host species specific pattern following previous findings [e.g. (Schaer *et al*., 2017)]. Sequences of the study are highlighted in bold blue. Numbers at nodes are ML bootstrap value (> 70) using 1000 replicates.

Haemosporidian parasite infections were limited to only 4 individuals of *E. helvum*, which correspond to a prevalence of 5.9% (4/68) in *E. helvum* and an overall prevalence of 4.2% (4/95). No infections were detected in the species *Nycteris* cf. *macrotis*, *Mops* cf. *condylurus* and *Mops* cf. *pumilus* (Tables 1 and S4). The molecular analysis identified the parasite infections in *E. helvum* as *Hepatocystis* parasites. Microscopic screening of the corresponding Giemsa-stained thin blood smears of the 4 PCR-positive samples did not result in detection of parasite stages which points to subpatent infections with a very low parasitaemia. The partial *cytb* sequences of *Hepatocystis* sp. of 2 samples (sample numbers KJ72, KJ82) were identical, the third sample (sample number J53) featured a few ambiguous base calls which point to a mixed haplotype infection but was otherwise identical to the other 2 samples. The *Hepatocystis* sequences are identical to published parasite sequences from fruit bat hosts in Nigeria and South Sudan (Schaer *et al*., 2017; Atama *et al*., 2019). The 4th sequence (sample number KJ63) featured about 96% identity with the other 3 sequences that were recovered from *Hepatocystis* parasite infections in *E. helvum* in this study. Interestingly, all 4 sequences showed higher similarities to parasite sequences from other bat host species than to the sole published sequence from *Hepatocystis* sp. from *E. helvum* in Gabon. The phylogenetic analysis that was performed to assess the phylogenetic relationships and possible geographic and/or host specific patterns of *Hepatocystis* parasites from Nigeria confirmed *Hepatocystis* as a monophyletic group and as sister clade to the rodent- and bat-infecting *Plasmodium (Vinckeia)* parasite clade as shown before (Fig. 2) (e.g. Galen *et al*., 2018). Within the African bat *Hepatocystis* clade, the *Hepatocystis* sequence from sample KJ63 groups basal, whereas the other 3 sequences fall within the main clade (Fig. 2). Overall, the *Hepatocystis* sp. sequences of different African bat species from several distant related African countries neither show geographic nor host specificity patterns confirming previous analysis (e.g. Schaer *et al*., 2017).

### Prevalence and phylogenetic characterization of *Trypanosoma* parasites

**Fig. 3.**
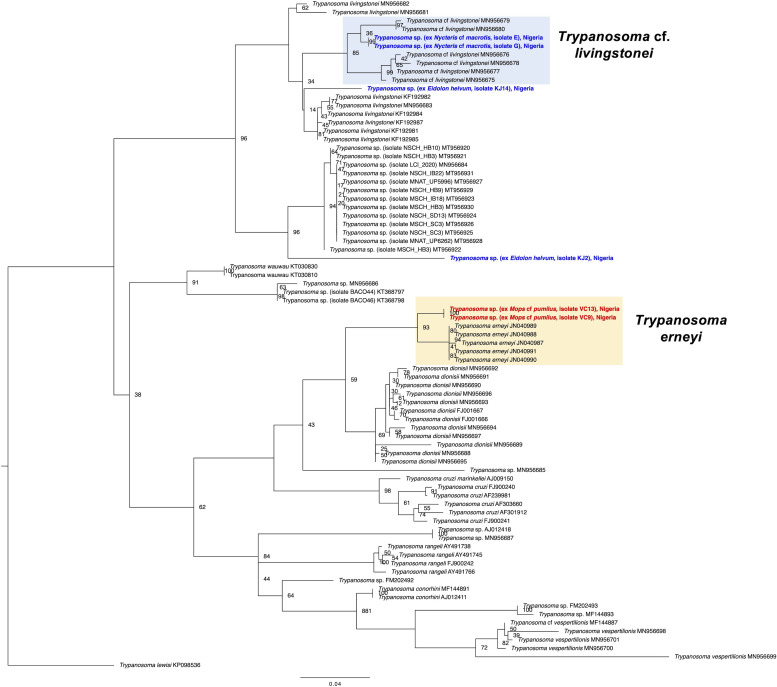
Phylogeny of the *Trypanosoma* parasites inferred by maximum likelihood analysis from the 18S rRNA gene (1022 bp) using TIM3 + I + G and *Trypanosoma lewisi* as outgroup. Numbers at nodes are ML bootstrap values using 10 000 replicates. The parasites from *Nycteris* cf. *macrotis* hosts of the study group closely with *T*. cf. *livingstonei* parasites. One sequence from the parasites from *E. helvum* hosts groups basal to *T. livingstonei* parasites, whereas the other one was recovered as separate lineage to *Trypanosoma* sp. parasites. The parasite sequences of *N.* cf. macrotis and *E. helvum* of the study are highlighted in bold blue, the parasite sequences of *Mops* cf. *pumilus* in red. The trypanosome sequences from *Mops* cf. *pumilus* hosts of the study group as distinct lineage to *T. erneyi* parasites with high support (bootstrap value 93).

**Fig. 4.**
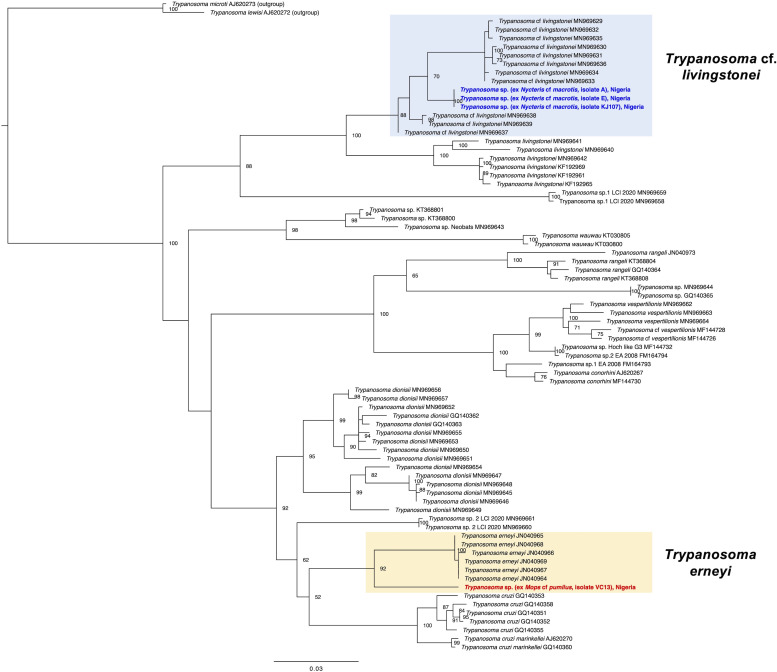
Phylogeny of the *Trypanosoma* parasites inferred by maximum likelihood analysis from the *gGAPDH* gene (894 bp) using GTR + I + G and *Trypanosoma lewisi* as outgroup. Numbers at nodes are ML bootstrap values using 10 000 replicates. The parasites from *Nycteris* cf. *macrotis* hosts of the study group closely with *T*. cf. *livingstonei* parasites supporting the results of the 18S rRNA analysis (the sequences of the study are highlighted in bold blue). The trypanosome sequence from *Mops* cf. *pumilus* of the study (highlighted in bold red) groups as distinct lineage to *T. erneyi* parasites with high support (bootstrap value 92).

Trypanosome infections were detected in 16 individuals from 3 out of the 4 bat species of the study (16/95, overall prevalence of 16.8%) (Tables 1 and S4). Two infections were confirmed in *E. helvum* (2/68, prevalence of 2.9%), 3 infections in *Mops* cf. *pumilus* (3/8, 37.5%) and a high prevalence of 68.8% was found in *Nycteris* cf. *macrotis* (11/16). No infections were recorded for 3 individuals of *Mops* cf. *condylurus* (Table 1). The trypanosomes of *Mops* cf. *pumilus* (Molossidae) were identified as species *Trypanosoma* cf. *erneyi* based on the blastn (https://blast.ncbi.nlm.nih.gov/) search of the 18S and *gGAPDH* sequences and the phylogenetic analysis. All 3 infected individuals (sample numbers VC6, VC9, VC13) featured identical 18S rRNA sequences which shared highest sequence identities (96.5%) with a reference sequence of a trypanosome from *Mops condylurus* in Mozambique (JN040989). One *gGAPDH* sequence was successfully generated (for sample number VC13), which showed highest identity (95.2%) with a trypanosome reference sequence from *Tadarida* sp. (Molossidae) again from Mozambique (JN04095). The species *Trypanosoma erneyi* has been described from bat species of the family Molossidae in Africa and the sequences of this study group closely to the reference sequences of *T. erneyi*, but in their own separate clade, in the 18S rRNA phylogenetic analysis as well as in the *gGAPDH* analysis (Figs 3 and 4). This finding indicates that the sequences of this study might represent a distinct species or subspecies, however with the lack of morphological information of these parasites (no parasite stages were detected in the blood smears), they are tentatively referred to as *T*. cf. *erneyi*.

The trypanosome infections of the host species *Nycteris* cf. *macrotis* belong to 1 parasite haplotype of *Trypanosoma* cf. *livingstonei*, all 18S rRNA as well as the *gGAPDH* sequences were identical. The 18S rRNA parasite haplotype showed highest identity (98.3%) with a trypanosome reference sequence from *Rhinolophus landeri* from Mozambique (KF192981), the *gGAPDH* parasite haplotype of *N.* cf. *macrotis* featured highest identity (94.1%) with a reference sequence from a trypanosome isolated from *Hipposideros caffer* in Mozambique (KF192969). The phylogenetic analysis of the 18S rRNA as well as the nuclear *gGAPDH* sequences grouped the representative parasite sequences of *N*. cf. *macrotis* within the *T.* cf. *livingstonei* clades that comprise sequences from *Nycteris* hosts among other bat species from Africa (Clement *et al*., 2020) (Figs 3 and 4). Unfortunately, the quality of the blood smears did not allow a further analysis of the detected parasite stages in 2 samples of *N*. cf. *macrotis* (Fig. S1).

Trypanosome infections were further detected in 2 individuals of *E. helvum.* The first sample (number KJ14) featured 97.5% nucleotide identity (18S rRNA) with a reference sequence of *T. livingstonei* (KF19298) from *Rhinolophus landeri* in Mozambique and in the ML analysis, the sequence groups basal to the *T. livingstonei* clade, though with low support (Fig. 3). The phylogenetic analysis of the second infected *E. helvum* (sample number KJ2) recovered the trypanosome sequence as basal to a clade that comprises *Trypanosoma* sp. sequences from African bats (bootstrap value of 96) that together group as sister clade to the clade that comprises *T.* cf. *livingstonei* and *T. livingstonei* (bootstrap value of 96) and might therefore represent a different species (Fig. 3). Unfortunately, no *gGAPDH* sequences for further characterization could be amplified for the 2 *Trypanosoma* infections of *E*. *helvum* and no parasite blood stages were detected in the blood smears.

## Discussion

This study adds information to the scarce knowledge about haemosporidian parasite infections in Nigerian bats. *Hepatocystis* parasites were detected in the straw-coloured fruit bat species *E. helvum*, which is only the second time that this bat species was recovered as host to *Hepatocystis*, the only other single evidence has been reported from Gabon (Boundenga *et al*., 2018). The *Hepatocystis* prevalence in *E. helvum* in this study (4 infected individuals, prevalence of 6%, 4/68) was quite low, compared to reports of *Hepatocystis* infections in African epauletted fruit bat species where prevalences can range from 25% up to about 90% (e.g. Schaer *et al*., 2013; Lutz *et al*., 2016; Schaer *et al*., 2017; Boundenga *et al*., 2018; Atama *et al*., 2019). The phylogenetic analysis recovered the *Hepatocystis* sequences from *E. helvum* within the African bat *Hepatocystis* clade without any apparent geographic or host species specific pattern following previous findings (e.g. Schaer *et al*., 2017). Yet, as the morphological verification of parasite stages in the blood of the vertebrate host (proof that a parasite has undergone asexual reproduction) is lacking for *Hepatocystis* parasites in *E. helvum* hosts, the findings must be considered with caution. In some cases, the sensitivity of PCR methods might verify haemosporidian parasite DNA (transmittable sporozoite stages) in wrong hosts, where the parasite life cycle has been aborted (Valkiunas *et al*., 2009). Interestingly, another African fruit bat species, *Rousettus aegyptiacus* that represents a true but perhaps rare host species of *Hepatocystis* (1 published record of a single infected individual) has been also documented from Nigeria (Atama *et al*., 2019). Systematic serial surveys of the 2 specific African fruit bat species *R. aegyptiacus* and *E. helvum* (Pteropodidae) along with the commonly infected epauletted bat host species are needed to understand the different prevalences and transmission dynamics of *Hepatocystis* infections in African bats. Both *R. aegyptiacus* and *E. helvum* are common fruit bat species that form large colonies in caves and trees, respectively, and feature an extensive distribution across sub-Saharan Africa (e.g. Cooper-Bohannon, 2016; Korine, 2016). With the reported bat host records, Nigeria might be a particular suitable country/area to investigate *Hepatocystis* infections in African fruit bats.

The trypanosome infections that were detected in the 3 bat species *E. helvum*, *Mops* cf. *pumilus* and *Nycteris* cf. *macrotis* belong to 2 or 3 different *Trypanosoma* species. The trypanosomes of *Mops* cf. *pumilus* (Molossidae) are related to *T. erneyi*, a species which is closely related to *T. dionisii* and *T. cruzi* and that has been described from different bat species exclusively of the family Molossidae in Africa (Lima *et al*., 2012). The parasites of the study might represent a subspecies of *T. erneyi* as the phylogenetic analysis recovered their sequences in their own separate clade (Figs 3 and 4). The trypanosome parasites of the host species *Nycteris* cf. *macrotis* are closely related to the lineage of *T*. cf. *livingstonei* parasites that has been reported from e.g. *Nycteris thebaica* bats from South Africa (Clement *et al*., 2020). *T*. cf. *livingstonei* infections appear to be common in different African insectivorous bat species and future investigations will determine whether the divergent lineage *T*. cf. *livingstonei* might represent a distinct species or a subspecies of *T. livingstonei* (Lima *et al*., 2013; Clement *et al*., 2020). Lacking the *gGAPDH* sequence for the trypanosome infections in the 2 individuals of *E. helvum* in this study did not allow an unambiguous assignment to 1 trypanosome species. One sample showed highest 18S rRNA nucleotide identity with *T. livingstonei*, whereas the other sample was recovered as basal to a clade that comprises *Trypanosoma* sp. sequences from different African bats, that are closely related to *T.* cf. *livingstonei* and *T. livingstonei* parasites (Fig. 3). The findings of this study confirm the notion that trypanosomes of African bats are phylogenetically diverse and might harbour a variety of yet undescribed species (Clement *et al*., 2020). Understanding the diversity and the phylogenetic relationships of bat trypanosomes are necessary to improve our understanding of the whole group of parasites that comprise species that are a threat to humans (Hamilton *et al*., 2009; Lima *et al*., 2012). Many trypanosome lineages of the *T. cruzi* clade might have evolved in African bat species (e.g. Lima *et al*., 2013; Clement *et al*., 2020) and therefore, a more targeted systematic sampling and a subsequent molecular characterization of trypanosome species from African bats is of particular importance.

## Data Availability

All *Hepatocystis* and trypanosome sequences of the study are available at GenBank (NCBI) with the accession numbers ON326584 – ON326587, ON332819 – ON332820, ON494560 – ON494563, ON571545 – ON571548.

## Supplementary material

For supplementary material accompanying this paper visit https://doi.org/10.1017/S0031182022000890. click here to view supplementary material
