# Supplementary material for: Molecular detection of Trypanosoma spp. and Hepatocystis parasite infections of bats in Northern Nigeria
Source: Parasitology. 2022 Jul 13;149(11):1460–7. doi: 10.1017/S0031182022000890 (PMC10090768; doi:10.1017/S0031182022000890)
Supplement: Supplementary file 1 [file S0031182022000890sup001.docx]

Molecular detection of *Trypanosoma* spp. and *Hepatocystis* parasite infections of bats in Northern Nigeria

Kamani J, Atuman YJ, Oche DA, Shekaro A, Werb O, Ejotre I, Schaer J

**Supplemental Material**

- **Supplementary Figure S1**. Micrographs of trypanosomes of the study
- **Supplemental Table S1:** Nucleotide primers used for parasite screening and sequencing
- **Supplemental Table S2:** GenBank accession numbers for phylogenetic analysis of *Hepatocystis* parasites
- **Supplementary Table S3**: GenBank accession numbers for *Hepatocystis* and trypanosome parasite sequences of the study
- **Supplementary Table S4:** Overview of investigated bat individuals of the study, parasite infections

**Supplemental Figure S1. Micrographs of trypanosomes.** *Trypanosoma* cf. *livingstonei* parasites detected in Giemsa-stained blood smears of *Nycteris* cf. *macrotis* bat hosts (a = sample I; b = sample no KJ2). Magnification of 1000x. Bars indicate 5µm. Due to the low quality of the blood smears, no assessment of the morphological characteristics was possible.

**Supplemental Table S1:** Nucleotide primers used for parasite screening and sequencing

| **Gene** | **Primer name** | **Sequence (5´- 3´)** | **Reference** |
| --- | --- | --- | --- |
| ***cytb***  (haemosporidian parasites) | Hep-F3 | CTTACCTTGGGGACAAATGAGTTATT | Schaer *et al*., 2013 |
|  | Hep-R3 | CTCTAGCACCAAATGTCATTTTAAATTG | Schaer *et al*., 2013 |
|  | DW2 | TAATGCCTAGACGTATTCCTGATTATCCAG | Perkins and Schall, 2002 |
|  | DW4 | TGTTTGCTTGGGAGCTGTAATCATAATGTG | Perkins and Schall, 2002 |
|  | 3932-F | GGGTTATGTATTACCTTGGGGTC | Perkins and Schall, 2002 |
|  | 3932-R | GACCCCAAGGTAATACATAACCC | Perkins and Schall, 2002 |
| ***cox1***  (haemosporidian parasites) | Cox1-F | CTATTTATGGTTTTCATTTTTATTTGGTA | Martinsen *et al*., 2008 |
|  | Cox1-R | AGGAATACGTCTAGGCATTACATTAAATCC | Martinsen *et al*., 2008 |
|  | Cox-in-F | ATGATATTTACARTTCAYGGWATTATTATG | Martinsen *et al*., 2008 |
|  | Cox-in-R | GTATTTTCTCGTAATGTTTTACCAAAGAA | Martinsen *et al*., 2008 |
|  | Cox-mid-F | TTATTCTGGTTTTTTGGTCATCCAG | Martinsen *et al*., 2008 |
|  | Cox-mid-R | CTGGATGACCAAAAAACCAGAATAA | Martinsen *et al*., 2008 |
| ***clpc***  (haemosporidian parasites) | Clpc-out-F | AAACTGAATTAGCAAAAATATTA | Martinsen *et al*., 2008 |
|  | Clpc-out-R | CGWGCWCCATATAAAGGAT | Martinsen *et al*., 2008 |
|  | Clpc-in-F | GATTTGATATGAGTGAATATATGG | Martinsen *et al*., 2008 |
|  | Clpc-in-R | CCATATAAAGGATTATAWG | Martinsen *et al*., 2008 |
| ***ef2***  (haemosporidian parasites) | EF2-F | GTTCGTGAGATCATGAACAAAAC | Schaer *et al*., 2013 |
|  | EF2-R | CCTTGTAAACCAGAACCAAA | Schaer *et al*., 2013 |
| **18S rRNA**  (trypanosomes) | TRY927F | GAAACAAGAAACACGGGAG | Noyes *et al*., 1999 |
|  | TRY927R | CTACTGGGCAGCTTGGA | Noyes *et al*., 1999 |
|  | SSU561F | TGGGATAACAAAGGAGCA | Noyes *et al*., 1999 |
|  | SSU561R | CTGAGACTGTAACCTCAAAGC | Noyes *et al*., 1999 |
| **gGAPDH**  (trypanosomes) | G5 | ACMAGRTCCACCACRCGGTG | Hamilton *et al*., 2004 |
|  | G3 | TTYGCCGYATYGGYCGCATGG | Hamilton *et al*., 2004 |
|  | G1 | CGCGGATCCASGGYCTYMTCGGBAMKGAGAT | Hamilton *et al*., 2004 |
|  | G4A | GTTYTGCAGSGTCGCCTTGG | Hamilton *et al*., 2004 |
| ***cytb***  (bat hosts) | L14724 | CGAAGCTTGATATGAAAAACCATCGTTG | Päabo 1989 |
|  | H15915 | GGAATTCATCTCTCCGGTTTACAAGAC | Irwin *et al*. 1991 |
| ***fgb***  (bat hosts) | FGB-F | CCACAACRGCATGTTCTTCAGCAC | Hassanin & Ropiquet, 2007 |
|  | FGB-R | GTATCTGCCATTTGGATTGGCTGC | Hassanin & Ropiquet, 2007 |
| ***acox2***  (bat hosts) | ACOX2-F1 | CCTSGGCTCDGAGGAGCAGAT | Salicini *et al*., 2011 |
|  | ACOX2-R1 | GGGCTGTGHAYCACAAACTCCT | Salicini *et al*., 2011 |

*cytb*, cytochrome b; *cox1*, cytochrome oxidase I; *clpc*, apicoplast caseinolytic protease; *ef2*, nuclear elongation factor 2; 18S rRNA*,* SSU rRNA, a component of the eukaryotic ribosomal small subunit; gGAPDH, Glyceraldehyde-3-phosphate dehydrogenase; Nuclear introns for bat genotyping: *acox*2 (Acyl-CoA oxidase 2, intron 3); *rogdi* (Rogdi-like protein gene, intron 7); *fgb* (Beta-fibrinogen gene, intron 7)

**References**

**Hamilton, P.B., Stevens, J.R., Gaunt, M.W., Gidley, J., Gibson, W.C**. 2004. Trypanosomes are monophyletic: evidence from genes for glyceraldehyde phosphate dehydrogenase and small subunit ribosomal RNA. *Int. J. Parasitol*. **34**, 1393–1404.

**Hassanin, A., Ropiquet, A.** 2007. Resolving a zoological mystery: the Kouprey is a real species, *Proc. R. Soc. Lond. B*. **274** (2007) 2849–2855.

**Irwin, D.M., Kocher, T.D., Wilson, A.C.,** 1991. Evolution of the Cytochrome *b* gene of mammals. *J. Mol. Evol.* **32**, 128 – 144.

**Martinsen, E.S., Perkins, S.L., Schall, J.,** 2008. A three-genome phylogeny of malaria parasites (*Plasmodium* and closely related genera): evolution of life-history traits and host switches. *Mol. Phylogenet. Evol.* **47**, 261–273.

**Noyes, H.A., Stevens, J.R., Teixeira, M., Phelan, J., Holz, P.** 1999. A nested PCR for the ssrRNA gene detects *Trypanosoma binneyi* in the *Platypus* and *Trypanosoma* sp. in wombats and kangaroos in Australia. *Int. J. Parasitol.* **29**, 331–39.

**Paäbo, S.,** 1989. Ancient DNA extraction, characterization, molecular cloning and enzymatic amplification. *PNAS* **86**, 1939–1943.

**Perkins, S.L., Schall, J.,** 2002. A molecular phylogeny of malarial parasites recovered from cytochrome *b* gene sequences. *J. Parasitol.* **88**, 972–978.

**Salicini, I., Ibanez, C., Juste, J**., 2011. Multilocus phylogeny and species delimitation within theNatterer's bat species complex in the Western Palearctic. *Mol. Phylogenet. Evol.* **61**, 888–898.

**Schaer, J., Perkins, S.L., Decher, J., Leendertz, F.H., Fahr, J., Weber, N., Matuschewski, K.,** 2013. High diversity of West African bat malaria parasites and a tight link with rodent *Plasmodium* taxa. *Proc. Natl. Acad. Sci. USA.* **110,** 17415-17419.

**Supplemental Table S2:** GenBank accession numbers for phylogenetic analysis of *Hepatocystis* parasites (samples from this study highlighted in bold)

| **Parasite (host group)** | **Sample (host species)** | ***cytb*** |
| --- | --- | --- |
| *Leucocytozoon* (Aves) | *Leucocytozoon* sp. (2109) | EU254518 |
|  | *Leucocytozoon* sp. (2208) | EU254520 |
|  | *Leucocytozoon* sp. (P157) | EU254519 |
| *Haemoproteus* (Aves) | *HHaemoproteus columbae* (2111) | EU254548 |
|  | *Haemoproteus columbae* (2146) | EU254553 |
|  | *Haemoproteus columbae* | FJ168562 |
| *Plasmodium* (Primates) | *Plasmodium falciparum* | DQ642845 |
|  | *Plasmodium gaboni* | FJ895307 |
|  | *Plasmodium reichenowi* | AJ251941 |
|  | *Plasmodium* sp. (ex *Pan troglodytes*) | HM235391 |
|  | *Plasmodium* sp. (ex *Gorilla gorilla*) | HM235288 |
| *Plasmodium* (Rodentia) | *Plasmodium berghei* | DQ414645 |
|  | *Plasmodium chabaudi* | DQ414649 |
|  | *Plasmodium vinckei* | DQ414651 |
|  | *Plasmodium yoelii* | AY099051 |
| *Plasmodium* (Chiroptera) | *Plasmodium cyclopsi* | KF159710 |
|  | *Plasmodium voltaicum* | KF159671 |
| *Polychromophilus* (Chiroptera) | *Polychromophilus* sp. (ex *Miniopterus villiersii*) | KF159681 |
|  | *Polychromophilus melanipherus* | JN990709 |
|  | *Polychromophilus murinus* | HM055583 |
|  | *Polychromophilus* sp. (ex *Pipistrellus* aff. *grandidieri*) | KF159714 |
| *Hepatocystis* (Chiroptera) | *Hepatocystis* sp. (ex *E. pusillus,* Guinea) | KF159683 |
|  | *Hepatocystis* sp. (ex *E. pusillus,* Guinea) | KF159680 |
|  | *Hepatocystis* sp. (ex *E. pusillus,* Guinea) | KF159693 |
|  | *Hepatocystis* sp. (ex *E. pusillus,* Guinea) | KF159704 |
|  | *Hepatocystis* sp. (ex *E. pusillus,* Guinea) | KF159683 |
|  | *Hepatocystis* sp. (ex *E. pusillus,* Cameroon) | MZ460922 |
|  | *Hepatocystis* sp. (ex *E. pusillus,* Cameroon) | MZ460918 |
|  | *Hepatocystis* sp. (ex *E. pusillus,* Cameroon) | MZ460915 |
|  | *Hepatocystis* sp. (ex *E. pusillus,* Nigeria) | MK634490 |
|  | *Hepatocystis* sp. (ex *E. pusillus,* Nigeria) | MK634507 |
|  | *Hepatocystis* sp. (ex *E. pusillus,* Nigeria) | MK634505 |
|  | *Hepatocystis* sp. (ex *E. pusillus,* Nigeria) | MK634501 |
|  | *Hepatocystis* sp. (ex *E. pusillus,* Nigeria) | MK634496 |
|  | *Hepatocystis* sp. (ex *E. pusillus,* Nigeria) | MK634503 |
|  | *Hepatocystis* sp. (ex *E. pusillus,* Nigeria) | MK634487 |
|  | *Hepatocystis* sp. (ex *E. pusillus,* Nigeria) | MK634497 |
|  | *Hepatocystis* sp. (ex *E. pusillus,* Nigeria) | MK634489 |
|  | *Hepatocystis* sp. (ex *E. pusillus,* Nigeria) | MK634506 |
|  | *Hepatocystis* sp. (ex *E. pusillus,* Nigeria) | MK634488 |
|  | *Hepatocystis* sp. (ex *E. pusillus,* South Sudan) | KY753527 |
|  | *Hepatocystis* sp. (ex *E. pusillus,* South Sudan) | KY753525 |
|  | *Hepatocystis* sp. (ex *Epom.* sp*.,* Kenya) | KY753518 |
|  | *Hepatocystis* sp. (ex *Epom.* sp*.,* Kenya) | KY753519 |
|  | *Hepatocystis* sp. (ex *Epom.* sp.*,* South Sudan) | KY753506 |
|  | *Hepatocystis* sp. (ex *Epom.* sp.*,* South Sudan) | KY753507 |
|  | *Hepatocystis* sp. (ex *Epom.* sp.*,* South Sudan) | KY753513 |
|  | *Hepatocystis* sp. (ex *Epom.* sp.*,* South Sudan) | KY753510 |
|  | *Hepatocystis* sp. (ex *Epom.* sp.*,* South Sudan) | KY753516 |
|  | *Hepatocystis* sp. (ex *E. buettikoferi*, Guinea) | KF159706 |
|  | *Hepatocystis* sp. (ex *E. buettikoferi*, Guinea) | KF159703 |
|  | *Hepatocystis* sp. (ex *E. franqueti*, Uganda) | KT750344 |
|  | *Hepatocystis* sp. (ex *E. franqueti*, Uganda) | KT750356 |
|  | *Hepatocystis* sp. (ex *E. franqueti*, Uganda) | KT750351 |
|  | *Hepatocystis* sp. (ex *E. franqueti*, Uganda) | KT750353 |
|  | *Hepatocystis* sp. (ex *E. franqueti*, South Sudan) | KY753503 |
|  | *Hepatocystis* sp. (ex *E. franqueti*, South Sudan) | KY753504 |
|  | *Hepatocystis* sp. (ex *Hipposideros* sp.*,* South Sudan) | KY753520 |
|  | *Hepatocystis* sp. (ex *H. monstrosus,* Liberia) | KF159689 |
|  | *Hepatocystis* sp. (ex *H. monstrosus,* Liberia) | KF159712 |
|  | *Hepatocystis* sp. (ex *H. monstrosus,* South Sudan) | KY753521 |
|  | *Hepatocystis* sp. (ex *M. leptodon,* Ivory Coast) | KF188066 |
|  | *Hepatocystis* sp. (ex *M. leptodon,* Liberia) | KF159705 |
|  | *Hepatocystis* sp. (ex *M. torquata,* Uganda) | KT750356 |
|  | *Hepatocystis* sp. (ex *M. torquata,* Uganda) | KT750357 |
|  | *Hepatocystis* sp. (ex *M. torquata,* Uganda) | KT750342 |
|  | *Hepatocystis* sp. (ex *N. veldkampii,* Guinea) | KF159698 |
|  | *Hepatocystis* sp. (ex *N. veldkampii,* Guinea) | EU254528 |
|  | *Hepatocystis* sp. (ex *N. veldkampii,* Liberia) | KF159698 |
|  | *Hepatocystis* sp. (ex *R. aegyptiacus,* Nigeria) | MK634508 |
|  | *Hepatocystis* sp. (*ex Eidolon helvum, Gabon)* | MG602649 |
|  | ***Hepatocystis* sp. (ex *Eidolon helvum*, J53, Nigeria)** | **ON494563** |
|  | ***Hepatocystis* sp. (ex *Eidolon helvum*, KJ72, Nigeria)** | **ON494561** |
|  | ***Hepatocystis* sp. (ex *Eidolon helvum*, KJ82, Nigeria)** | **ON494560** |
|  | ***Hepatocystis* sp. (ex *Eidolon helvum*, KJ63, Nigeria)** | **ON494562** |

Mozam. = Mozambique, Switz. = Switzerland; SL = Sierra Leone, SoSu = South Sudan

**Supplemental Table S3**: GenBank accession numbers for *Hepatocystis* and trypanosome parasite sequences of the study

| **Sample number** | **Bat species*** | ***cytb* (*Hepatocystis*)** | **18S rRNA (trypanosome)** | **gGAPDH (trypanosome)** |
| --- | --- | --- | --- | --- |
| J53 | *Eidolon helvum* | ON494563 | NA | NA |
| KJ2 | *Eidolon helvum* | NA | ON326584 | - |
| KJ14 | *Eidolon helvum* | NA | ON326585 | - |
| KJ63 | *Eidolon helvum* | ON494562 | NA | NA |
| KJ72 | *Eidolon helvum* | ON494561 | NA | NA |
| KJ82 | *Eidolon helvum* | ON494560 | NA | NA |
| VC9 | *Mops* cf*. pumilus* | NA | ON332819** | - |
| VC13 | *Mops* cf*. pumilus* | NA | ON332820** | ON571548 |
| A | *Nycteris* cf*. macrotis* | NA | - | ON571545*** |
| E | *Nycteris* cf*. macrotis* | NA | ON326586*** | ON571546*** |
| G | *Nycteris* cf*. macrotis* | NA | ON326587*** | - |
| KJ107 | *Nycteris* cf*. macrotis* | NA | - | ON571547*** |

*Please note that almost all 95 bat individuals have been genotyped; **18sRNA sequences identical for all infected *M*. cf. *pumilus*; ***all eleven infected *N*. cf. *macrotis* featured identical trypanosome 18sRNA and gGAPDH sequences; NA = not applicable (no infection with the respective parasite taxon); - = sequence could not be successfully amplified

**Supplemental Table S4:** Overview of investigated bat individuals of the study, parasite infections

| **Sample**  **Number** | **Bat species** | **Infection with**  **haemosporidian parasite** | **Infection with trypanosome** |
| --- | --- | --- | --- |
| J201 | *Eidolon helvum* | - | - |
| J204 | *Eidolon helvum* | - | - |
| J206 | *Eidolon helvum* | - | - |
| J208 | *Eidolon helvum* | - | - |
| J210 | *Eidolon helvum* | - | - |
| J216 | *Eidolon helvum* | - | - |
| J224 | *Eidolon helvum* | - | - |
| J231 | *Eidolon helvum* | - | - |
| J233 | *Eidolon helvum* | - | - |
| J239 | *Eidolon helvum* | - | - |
| J240 | *Eidolon helvum* | - | - |
| J242 | *Eidolon helvum* | - | - |
| J25 | *Eidolon helvum* | - | - |
| J40 | *Eidolon helvum* | - | - |
| J50 | *Eidolon helvum* | - | - |
| J51 | *Eidolon helvum* | - | - |
| J53 | *Eidolon helvum* | *Hepatocystis* sp. | - |
| J55 | *Eidolon helvum* | - | - |
| J56 | *Eidolon helvum* | - | - |
| J57 | *Eidolon helvum* | - | - |
| J58 | *Eidolon helvum* | - | - |
| J59 | *Eidolon helvum* | - | - |
| J61 | *Eidolon helvum* | - | - |
| J62 | *Eidolon helvum* | - | - |
| J63 | *Eidolon helvum* | - | - |
| J64 | *Eidolon helvum* | - | - |
| J65 | *Eidolon helvum* | - | - |
| J66 | *Eidolon helvum* | - | - |
| J67 | *Eidolon helvum* | - | - |
| J68 | *Eidolon helvum* | - | - |
| J82 | *Eidolon helvum* | - | - |
| KJ1 | *Eidolon helvum* | - | - |
| KJ10 | *Eidolon helvum* | - | - |
| KJ11 | *Eidolon helvum* | - | - |
| KJ111 | *Eidolon helvum* | - | - |
| KJ12 | *Eidolon helvum* | - | - |
| KJ121 | *Eidolon helvum* | - | - |
| KJ125 | *Eidolon helvum* | - | - |
| KJ126 | *Eidolon helvum* | - | - |
| KJ127 | *Eidolon helvum* | - | - |
| KJ129 | *Eidolon helvum* | - | - |
| KJ13 | *Eidolon helvum* | - | - |
| KJ130 | *Eidolon helvum* | - | - |
| KJ131 | *Eidolon helvum* | - | - |
| KJ132 | *Eidolon helvum* | - | - |
| KJ14 | *Eidolon helvum* | - | *Trypanosoma* cf. *livingstonei* |
| KJ15 | *Eidolon helvum* | - | - |
| KJ16 | *Eidolon helvum* | - | - |
| KJ17 | *Eidolon helvum* | - | - |
| KJ18 | *Eidolon helvum* | - | - |
| KJ19 | *Eidolon helvum* | - | - |
| KJ2 | *Eidolon helvum* | - | *Trypanosoma* cf. *livingstonei* |
| KJ21 | *Eidolon helvum* | - | - |
| KJ3 | *Eidolon helvum* | - | - |
| KJ4 | *Eidolon helvum* | - | - |
| KJ5 | *Eidolon helvum* | - | - |
| KJ6 | *Eidolon helvum* | - | - |
| KJ63 | *Eidolon helvum* | *Hepatocystis* sp. | - |
| KJ69 | *Eidolon helvum* | - | - |
| KJ7 | *Eidolon helvum* | - | - |
| KJ70 | *Eidolon helvum* | - | - |
| KJ72 | *Eidolon helvum* | *Hepatocystis* sp. | - |
| KJ8 | *Eidolon helvum* | - | - |
| KJ82 | *Eidolon helvum* | *Hepatocystis* sp. | - |
| KJ87 | *Eidolon helvum* | - | - |
| KJ9 | *Eidolon helvum* | - | - |
| KJ90 | *Eidolon helvum* | - | - |
| KJ92 | *Eidolon helvum* | - | - |
| GB118 | *Mops* cf. *condylurus* | - | - |
| GB119 | *Mops* cf. *condylurus* | - | - |
| GB120 | *Mops* cf. *condylurus* | - | - |
| VC11 | *Mops* cf. *pumilus* | - | - |
| VC12 | *Mops* cf. *pumilus* | - | - |
| VC17 | *Mops* cf. *pumilus* | - | - |
| VC3 | *Mops* cf. *pumilus* | - | - |
| VC5 | *Mops* cf. *pumilus* | - | - |
| VC6 | *Mops* cf. *pumilus* | - | *Trypanosoma* cf*. erneyi* |
| VC9 | *Mops* cf. *pumilus* | - | *Trypanosoma* cf*. erneyi* |
| VC13 | *Mops* cf. *pumilus* | - | *Trypanosoma* cf*. erneyi* |
| KJ-A | *Nycteris* cf. *macrotis* | - | *Trypanosoma* cf. *livingstonei* |
| KJ-B | *Nycteris* cf. *macrotis* | - | *Trypanosoma* cf. *livingstonei* |
| KJ-C | *Nycteris* cf. *macrotis* | - | *Trypanosoma* cf. *livingstonei* |
| KJ-D | *Nycteris* cf. *macrotis* | - | - |
| KJ-E | *Nycteris* cf. *macrotis* | - | *Trypanosoma* cf. *livingstonei* |
| KJ-F | *Nycteris* cf. *macrotis* | - | *Trypanosoma* cf. *livingstonei* |
| KJ-G | *Nycteris* cf. *macrotis* | - | *Trypanosoma* cf. *livingstonei* |
| KJ-H | *Nycteris* cf. *macrotis* | - | - |
| KJ-I | *Nycteris* cf. *macrotis* | - | *Trypanosoma* cf. *livingstonei* |
| KJ-J | *Nycteris* cf. *macrotis* | - | - |
| KJ-K | *Nycteris* cf. *macrotis* | - | *Trypanosoma* cf. *livingstonei* |
| KJ104 | *Nycteris* cf. *macrotis* | - | *Trypanosoma* cf. *livingstonei* |
| KJ107 | *Nycteris* cf. *macrotis* | - | *Trypanosoma* cf. *livingstonei* |
| KJ128 | *Nycteris* cf. *macrotis* | - | *Trypanosoma* cf. *livingstonei* |
| KJ-L | *Nycteris* cf. *macrotis* | - | - |
| KJ-M | *Nycteris* cf. *macrotis* | - | - |
